# High-power short-duration ablation index–guided pulmonary vein isolation protocol using a single catheter

**DOI:** 10.1007/s10840-022-01226-9

**Published:** 2022-05-20

**Authors:** Patrick Badertscher, Sven Knecht, Florian Spies, Gian Völlmin, Beat Schaer, Nicolas Schärli, Flurina Bosshard, Stefan Osswald, Christian Sticherling, Michael Kühne

**Affiliations:** 1 Department of Cardiology, University Hospital Basel, Petersgraben 4, 4031 Basel, Switzerland; 2 Cardiovascular Research Institute Basel, University Hospital Basel, Petersgraben 4, 4031 Basel, Switzerland

**Keywords:** Atrial fibrillation, Catheter ablation, Efficacy

## Abstract

**Background:**

Catheter ablation for atrial fibrillation (AF) is the most commonly performed electrophysiological procedure. To improve healthcare utilization, we aimed to compare the efficacy, efficiency, and safety of a minimalistic, streamlined single catheter ablation approach using a high-power short-duration ablation index–guided protocol (HPSD) vs. a control single-catheter protocol (SP).

**Methods:**

Pulmonary vein isolation (PVI) with a single transseptal puncture without a multipolar mapping catheter was performed in 91 patients. Left atrial mapping was performed with the ablation catheter, only. Pacing maneuvers were used to confirm exit block. Procedural characteristics and success rates were compared using HPSD (*n* = 34) vs. a control (*n* = 57) ablation protocol. Freedom from recurrence was defined as a 1-year absence of AF episodes > 30 s, beyond the 3-month blanking period.

**Results:**

Using the HPSD protocol the median procedure and RF ablation time were significantly shorter compared to the SP, 84 (IQR 76–100) vs. 118 min (IQR 104–141) and 1036 (898–1184) vs. 1949s (IQR 1693–2261), respectively, *p* < .001 for all. First-pass PVI was achieved using the HPSD protocol in 88% and using the SP in 87% of patients, *p* = 1.0. No procedural complications were observed. High-sensitivity cardiac troponin levels were significantly higher in patients using the HPSD protocol compared to the SP. At 12 months follow-up, 87% patients remained free from AF with no differences between groups.

**Conclusions:**

A minimalistic, HPSD ablation index–guided PVI with a single-catheter approach is very efficient, safe, and associated with excellent clinical outcomes at 1 year.

**Supplementary Information:**

The online version contains supplementary material available at 10.1007/s10840-022-01226-9.

## Introduction

Pulmonary vein isolation (PVI) is the cornerstone of the treatment of atrial fibrillation (AF) [1]. Based on the ground-breaking study by Haissaguerre et al., a dedicated circular mapping catheter (CMC) was developed to detect the conduction into the PV and to confirm the electrical isolation of the PVs from the left atrium (LA) after circumferential radiofrequency (RF) ablation [2]. Since then, the use of a multipolar catheter such as a CMC or other high-density mapping catheter has been the widely accepted means for assessment of conduction breakthrough from the PVs to the LA and vice versa. To simplify and speed up the procedure compared to the established point-by-point focal RF ablation, balloon-based technologies such as cryoballoon devices were developed [3–5]. The procedural simplicity of these “single-shot” technologies led to shorter procedure times without the need of additional diagnostic catheters for the confirmation of PVI. [6, 7]

Nonetheless, current balloon technologies are mostly limited to PV ablation. As the RF PVI procedure evolved over the years with the advent of contact-force sensing ablation catheters [8–10] in combination with efficacy parameters such as the ablation or lesion size index [11–13], high efficacy of first-pass isolations between 34 and 84% were reported [12–14]. This efficacy was increased by implementing standardized high-power short-duration (HPSD) ablation protocols in combination with defined lesion distances implemented in the CLOSE protocol [15]. While multipolar catheters remain valuable tools for complex atrial macro-re-entrant tachycardias after PVI and for redo procedures to facilitate the localization conduction gaps [16], they may not be necessary in first-time PVI procedures. Previous studies have demonstrated that a single-tip catheter approach was capable of assessing PVI via pacing maneuvers to confirm exit block and to localize conduction gaps [17–19]. The use of a single catheter also obviates a second transseptal puncture or the exchange of mapping and ablation catheters with a single transseptal puncture, respectively.

Thus, the purpose of the current study was to compare the efficacy, efficiency, and safety of a minimalistic, streamlined single-catheter RF ablation approach using a HPSD ablation index–guided protocol with a control single-catheter RF ablation protocol (SP) for first-time PVI in patients with AF.

## Methods

### Study population

Consecutive patients from the SWISS-AF-PVI registry with PVI using a force-sensing catheter in combination with a 3D electroanatomic mapping system (EAM) (Carto3, Biosense Webster; USA) system were included in the study. All patients gave written informed consent, and the study was approved by the local ethics committee. All patients underwent single-catheter PVI. The first 57 patients (63%) undergoing PVI using the SP were compared to the following 34 patients (37%) undergoing PVI using the HPSD ablation index–guided protocol. The authors had full access to and take full responsibility for the integrity of the data.

### Mapping and ablation protocol

After transesophageal echocardiography to rule out LA thrombus, ablations were performed under conscious sedation using midazolam and fentanyl based on a standardized protocol. Briefly, the ablation catheter was advanced under the guidance of the 3D EAM system in a right anterior oblique and left anterior oblique view in the right atrium and subsequently in the coronary sinus vein as an anatomical landmark for transseptal puncture. This was performed under fluoroscopic guidance. Pre-procedural cardiac magnetic resonance imaging was reconstructed and imported in the 3D EAM System (Carto3, Biosense Webster, Diamond Bar, California, USA) and pre-registered based on the ablation catheter in the coronary sinus vein. Fast anatomical mapping was performed under respiration compensation using the ablation catheter with a resolution of 14. Precise delimitation of the ostia of the pulmonary veins was performed using the “swing-fall” technique as previously described [19]. As we pulled the catheter back from the veins into the atrium, we inserted a tag precisely where the catheter force vector “swings” and/ or the tip of the catheter abruptly “falls” into the atrium. Concomitant prior atrial flutter was documented in 22 patients (24%), and these patients underwent cavotricuspid isthmus ablation (CTI) after PVI. Additional CTI ablation was performed in 14 patients (25%) in the HPSD ablation index–guided protocol and 8 patients (24%) PVI using the SP. While the RF time is reported separately for PVI and the CTI ablation, all other procedural characteristics are reported for PVI and CTI combined in these patients.

### HPSD ablation index–guided protocol vs. control ablation protocol

Ablation was performed at the anatomical ostium defined by the FAM using the ablation index with a minimal target value of 400 at the posterior wall and of 450 at the anterior wall [20]. Interlesion distance was set to 6 mm. For the HPSD protocol (HPSD group), power was set to 35 W at the posterior wall and 50 W at the anterior wall, and for the SP group, power was limited to 25 W at the posterior wall and 30 W at the anterior wall in a power controlled mode. All ablations were performed using the Biosense Thermocool Surround Flow SF® ablation catheter with a flow rate of 17 ml/min.

### Documentation of entrance and exit block

**Fig. 1:**
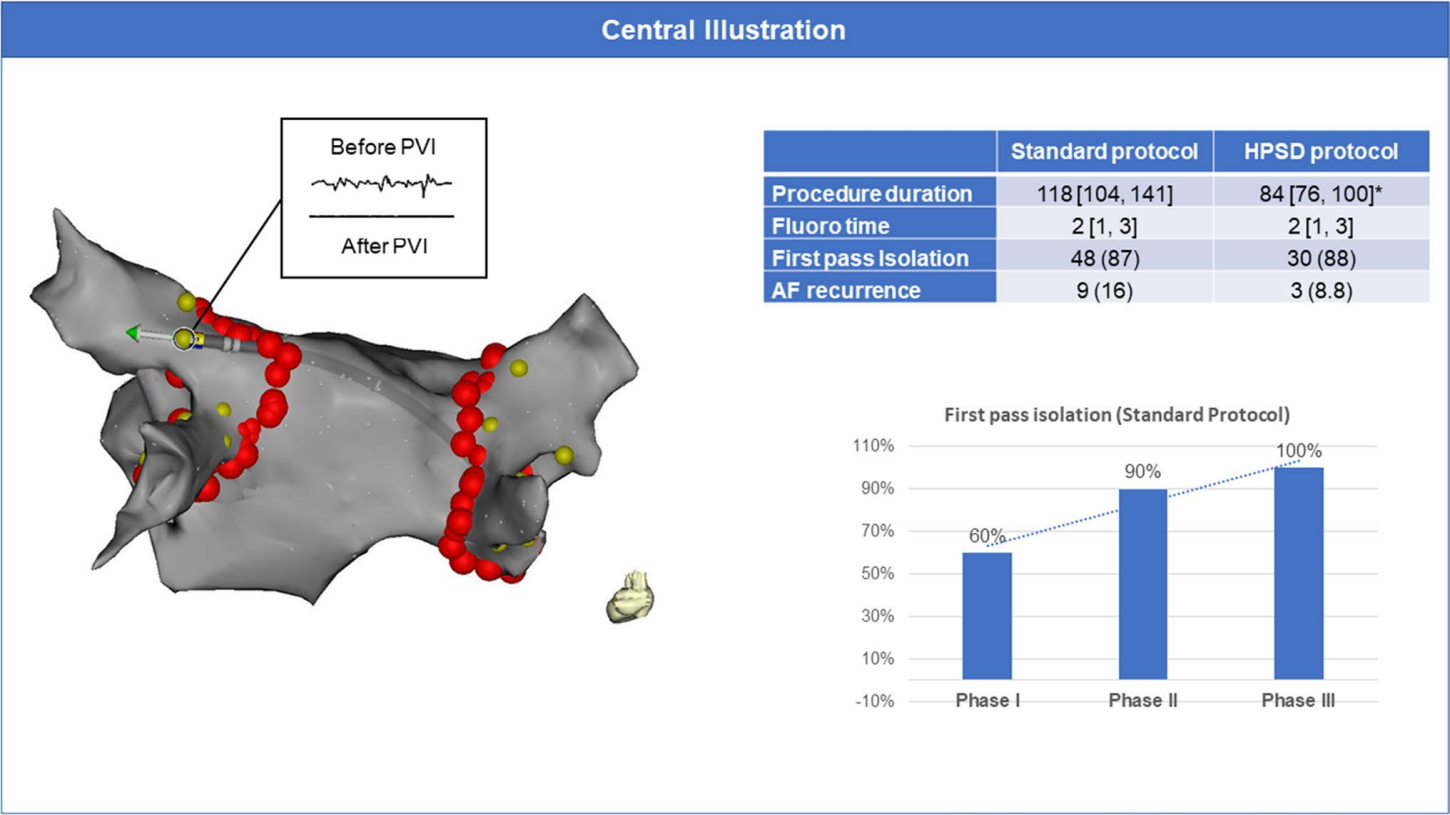
Efficacy and safety of a high-power short-duration ablation index–guided protocol for pulmonary vein isolation using a single catheter**. Left**: Documentation of entrance and exit block using a single catheter: After anatomical circumferential ablation around the pulmonary veins (red tags), the ablation catheter was positioned (with adequate contact force displayed by the orientation of the force vector) distal to the circumferential lesion to check for local signals. If no signals were identified (entrance block), pacing for exit block from the vein was performed at the anatomical ostium of every vein at four evenly distributed locations (yellow tags) in each pulmonary vein by pacing with 10 V and a pulse width of 1.5 ms. Right, top: Outcome measures for a control ablation protocol using a single catheter versus a high-power short-duration ablation index–guided protocol for PVI using a single catheter. * represents a *p* value of < 0.05. Right, bottom: To account for a potential learning effect during implementation of the single-catheter protocol, we arbitrarily split the control ablation protocol group into three consecutive groups (Phases I–III). Over time, the amount of first-pass isolation significantly increased (*p* = 0.01) from 60% after 11 pts to 100% after 41 cases

If the patient was in AF after closing the circumferential lesion set, cardioversion was performed. The ablation catheter was then positioned (with adequate contact force, defined as ≥ 10 g) distal to the circumferential lesion to check for local signals. If no signals were identified (entrance block), pacing for exit block from the vein was performed at the anatomical ostium of every vein at four evenly distributed locations by pacing with 10 V and a pulse width of 1.5 ms (Fig. 1).

When conduction was observed, the localization of the conduction gap was identified at the earliest recorded pulmonary vein potential (PVP) by placing the ablation catheter within the circumferential ablation line. Neither a fixed waiting period after confirmation of PVI nor application of adenosine or isoproterenol to check for dormant conduction was performed.

### Use of a circular mapping catheter to verify PVI

To account for a potential learning effect during implementation of the single-catheter protocol, we prospectively split the 57 patients of the SP group into three consecutive groups: in the first group of 11 patients, a circular mapping catheter was used to verify PVI after the confirmation using the single catheter approach (phase I), a group of 30 patients without CMC (phase II), and a third group of 16 patients with CMC to verify PVI after the single-catheter approach (phase III). In phases I and III, it should be noted that the CMC was not used for fast anatomical mapping at the beginning of the procedure, only for confirmation of PVI before sheath removal.

### Biomarkers of myocardial injury

Blood samples were collected in a fasting state on the morning before the procedure and 24 h after the procedure. A hs-cTnT assay (Roche Elecsys 2010 high-sensitivity troponin T, Roche Diagnostics) with a 99th percentile concentration of 14 ng/L with a corresponding coefficient of variation of 10% at 13 ng/L was used. [21].

### Endpoints and follow-up

The primary (efficacy) endpoint of the study was freedom from recurrence during 1-year follow-up. After a blanking period of 3 months, any documentation of an AF or atrial tachycardia (AT) episode lasting more than 30 s was counted as recurrence. Secondary endpoints (efficiency) were the procedural duration from puncture of the groin to removal of the sheath (procedure duration), stick to map time, duration of the fast anatomical mapping of the LA (Map duration), duration of the RF energy delivery from first to last application of RF energy (RF duration), cumulative radiofrequency energy duration (RF time), and isolation of the PV during first encirclement (first-pass isolation (FPI)). Follow-up was performed at 3, 6, and 12 months including a detailed history, physical examination, ECG, and 7-day Holter monitoring. In case of symptomatic recurrence outside these planned outpatient clinic visits, a 12-lead ECG or Holter ECG was performed to document the arrhythmia. For the purpose of this study, major complications (safety) were defined, according to the recent HRS/EHRA expert consensus statement of CA or surgical ablation of AF [22], as complications that result in prolongation of hospital stay or another hospitalization, those that require additional intervention for treatment, and/or those that result in significant injury or death.

### Statistical analysis

Continuous variables are presented as mean ± one standard deviation and median. For continuous variables, comparisons were made using Student’s *T* test or Mann–Whitney *U* test. Test for normality was performed using the Kolmogorov–Smirnov test. Discrete variables were compared using Fisher’s exact test. Survival analysis was conducted using Kaplan–Meier curves. Difference in time-to-event stratification was tested by the use of the log-rank test. All analyses were performed using SPSS (version 22.0, SPSS Inc., Chicago, ILL) and a *p* value < 0.05 was considered statistically significant.

## Results

### Study population

A total of 91 patients undergoing AF ablation using the single-catheter approach were analyzed. Thirty-four patients (37%) were ablated using a HPSD ablation index–guided protocol and 57 patients (63%) were ablated using a SP using a single catheter for PVI.

### Patient demographics

The baseline patient characteristics are summarized in Table 1. Median age was 62 years (IQR 57–70) and 69% of patients were male. Fifty-nine patients (65%) had paroxysmal AF, and 32 (35%) had persistent AF. No patient had long-standing persistent AF. The median CHA_2_DS_2_VASc score was 1 (IQR 1–2) and the median EHRA score was 2 (IQR 2–3). Median LA diameter was 40 mm (IQR 38–43) and LAVI was 36 ml/m^2^ (IQR 28–46). Concomitant atrial flutter was documented in 22 patients (24%) and 5 patients (5%) had a history of CTI ablation. No statistical differences in baseline characteristics were observed between the HPSD ablation-index guided protocol group and the SP group.

**Table 1:** Baseline characteristics of the patients

	**All patients (*n* = 91)**	**Control protocol (*n* = 57)**	**HPSD protocol (*n* = 34)**	***p* value**
Age (median [IQR])	62 [57, 70]	60 [56, 69]	63 [59, 70]	0.2
Male (%)	63 (69)	42 (74)	21 (62)	0.339
BMI (median [IQR])	26 [24, 29]	26 [24, 29]	26 [23, 29]	0.896
CHA2DS2-VASc (median [IQR])	1 [1, 2]	1 [1, 2]	2 [1, 3]	0.438
EHRA score (median [IQR])	2 [2, 3]	2 [2, 3]	2 [2]	0.274
Persistent AF (%)	32 (35)	19 (33)	13 (38)	0.805
Atrial flutter documented (%)	20 (22)	12 (21)	8 (24)	0.989
History of CTI ablation (%)	5 (5)	4 (7)	1 (3)	0.726
LVEF (median [IQR])	60 [55, 66]	61 [58, 66]	59 [54, 65]	0.115
LA (median [IQR])	40 [38, 43]	40 [38, 43]	41 [37, 43]	0.853
LAVI (median [IQR])	36 [28, 46]	38 [30, 47]	34 [28, 41]	0.137
CAD (%)	5 (5)	3 (5)	2 (6)	1
History of MI (%)	6 (7)	4 (7)	2 (6)	1
History of valve surgery (%)	2 (2)	2 (4)	0 (0)	0.715
History of stroke (%)	2 (2)	2 (4)	0 (0)	0.715
History of heart failure (%)	2 (2)	2 (4)	0 (0)	0.715
OSA (%)	7 (8)	4 (7)	3 (9)	1
HTN (%)	48 (53)	30 (53)	18 (53)	1
DM (%)	3 (3)	0 (0)	3 (9)	0.094
PAD (%)	2 (2)	2 (4)	0 (0)	0.715
Renal failure (%)	4 (4)	3 (5)	1 (3)	1
Hyperthyroidism (%)	1 (1)	1 (2)	0 (0)	1
Hypothyroidism (%)	4 (4)	4 (7)	0 (0)	0.293
Smoking (%)	39 (43)	23 (40)	16 (47)	0.684

Data are presented as *n* (%) or median (IQR). Abbreviations: *IQR* interquartile range; *BMI* body mass index; *EHRA* European Heart Rhythm Association; *CTI* cavotricuspid isthmus ablation; *LVEF* left ventricular ejection fraction; *LA* left atrium; *LAVI* left atrium volume indexed; *CAD* coronary artery disease; *MI* myocardial infarction; *OSA* obstructive sleep apnea; *HTN* hypertension; *DM* diabetes mellitus; *PAD* peripheral artery disease; *hs-cTn* high-sensitivity cardiac troponin

### Procedural characteristics and biomarkers of myocardial injury

**Fig. 2:**
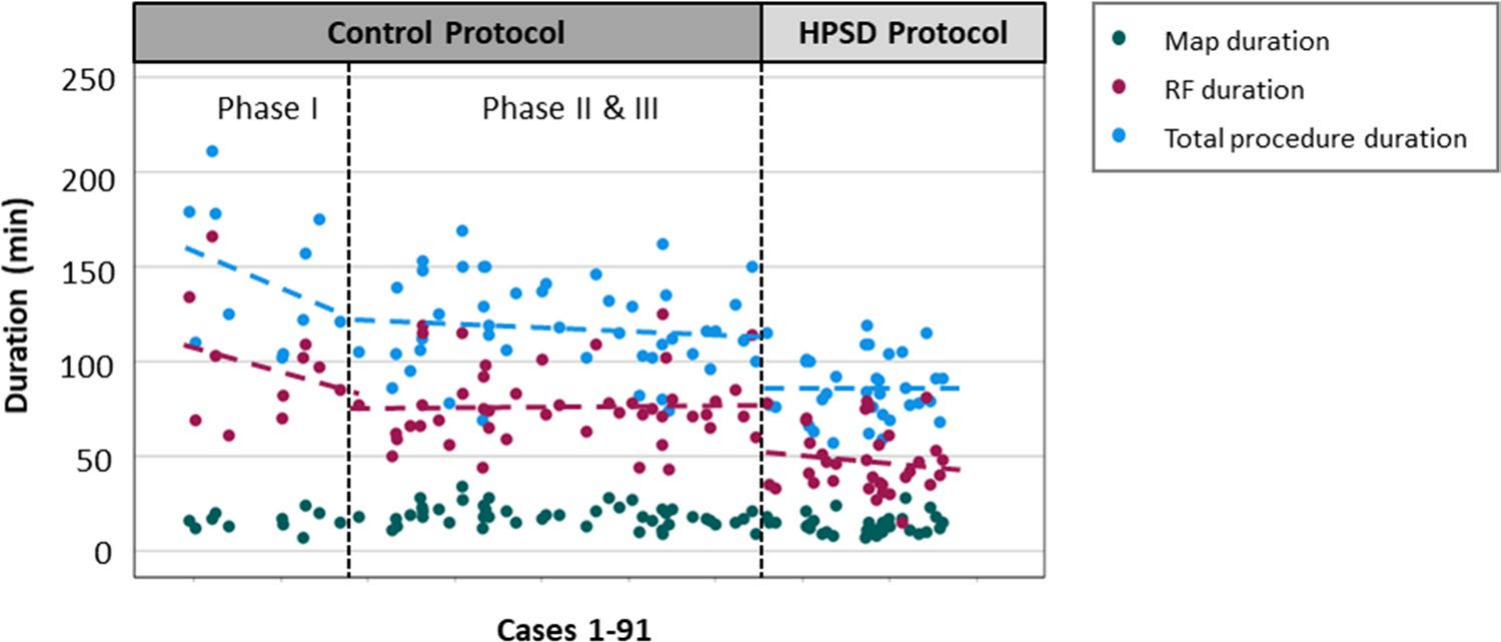
Simple scatter plot representing map duration, ablation duration, and total procedure duration over time. Map duration (blue dots), ablation (RF) duration (red dots), and total procedure duration (green dots) are plotted over a time line (x-axis). The duration in minutes is represented on the y-axis. The cohort was divided into 3 groups: a training phase (phase I), a stabilized phase of the control protocol (phases II and III), and the interventional phase performing the HPSD ablation. The validity of this stratification was confirmed by the individual and selective linear regression modelling with steep slopes in phase 1 (b1 − 0.108 and − 0.072 for the procedure duration and RF duration, respectively) and a horizontal course of the data for the stable phases II and III and HPSD (b1 of 0.002 and − 0.023 for the RF duration and − 0.010 and − 0.001 for the procedure duration, respectively)

Table 2 provides a summary of the procedural data. In brief, in the HPSD group the median procedure time was 84 min (IQR 76–100), median map time was 12 min (IQR 10–16), and RF ablation time was 1036 s (898–1184). These times were significantly shorter than the median procedure duration and RF ablation time seen in the SP group (118 min (IQR 104–141) and 1949s (IQR 1693–2261), respectively, *p* < 0.001 for all). Median fluoroscopy time and median radiation exposure were minimal and similar between both groups. Total RF energy delivered was significantly lower in the HSPD group compared to the SP group despite identical acute success of PVI (43,312 Joules (J) (IQR 37,484–53,156 J vs. 54,565 J (IQR 48,917–61,174 J), *p* < 0.001). Findings were confirmed when excluding 11 patients from the first “training” phase of the SP group (Table S1). To assess the impact of a potential training effect, the SP group was investigated in three consecutive groups. Over time, procedure duration decreased mainly due to reduction of RF duration and the amount of FPI significantly increased (Fig. 1, central illustration**).** The HPSD protocol using a single-catheter approach was implemented after the SP and thus was not assessed in different groups for a training effect, but map, ablation, and procedural duration decreased over time (Fig. 2).

**Table 2:** Procedural characteristics of the patients

	**All patients (*n* = 91)**	**Control protocol (*n* = 57)**	**HPSD protocol (*n* = 34)**	***p* value**
RF time PVI (sec, median [IQR])	1654 [1130, 2024]	1949 [1693, 2261]	1036 [898, 1184]	** < 0.001**
Fluoro time (min, median [IQR])	2 [1, 3]	2 [1, 3]	2 [1, 3]	0.7
Fluoro dose (Gycm^2^, median [IQR])	168 [76, 341]	215 [79, 410]	149 [69, 205]	0.215
Procedure duration (min, median [IQR])	105 [85, 127]	118 [104, 141]	84 [76, 100]	** < 0.001**
Stick to map time (min, median [IQR])	19 [13, 26]	20 [13, 28]	18 [14, 22]	0.19
Map duration (min, median [IQR])	16 [12, 20]	18 [15, 21]	12 [10, 16]	** < 0.001**
RF duration (min, median [IQR])	69 [48, 80]	75 [66, 97]	44 [35, 57]	** < 0.001**
Number of lesions	79 [67, 91]	85 [75, 105]	67 [60,80]	** < 0.001**
First-pass isolation (%)	78 (88)	48 (87)	30 (88)	1
CTI ablation (%)	22 (24)	14 (25)	8 (24)	1
RF time for CTI (sec, median [IQR])	312 [244, 526]	298 [255, 534]	335 [209, 501]	0.733
Overall RF time (sec, median [IQR])	1742 [1248, 2124]	2006 [1889, 2373]	1107 [934, 1329]	** < 0.001**
Energy dose (Joules, median [IQR])	52,156 [42630, 59347]	54,565 [48917, 61174]	43,312 [37484, 53156]	** < 0.001**
hs-cTn before RF (ng/L, median [IQR])	7 [5, 11]	7 [5, 11]	7 [6, 11]	0.618
hs-cTn after RF (ng/L, median [IQR])	864 [698, 1125]	823 [663, 1020]	996 [724, 1264]	**0.029**
***Outcome data***				
Recurrence during FU (%)	12 (13)	9 (16)	3 (8.8)	0.669
Redo procedures (%)	5 (5.5)	5 (8.8)	0 (0)	1

Data are presented as *n* (%) or median (IQR). Patients from the training phase (*n* = 11) in the control protocol were excluded for this analysis. Abbreviations: *IQR* interquartile range; *RF* radiofrequency energy; *Fluoro* fluoroscopy; *CTI* cavotricuspid isthmus ablation; *FU* follow-up

While hs-cTnT levels before the procedure were similar between both groups (7 ng/l (IQR 5–11 ng/L) vs. 7 ng/l (IQR 6–11 ng/l), hs-cTnT levels were elevated in all patients after the ablation (864 ng/l, IQR 695–1140 ng/L). hs-cTnT levels after ablation were significantly higher in patients in the HSPD group compared to the SP group (996 ng/L (IQR 724–1264) vs. 828 ng/l (IQR 636–1023), *p* = 0.037).

### Confirmation of entrance and exit block

In all patients, entrance and exit blocks were confirmed using solely the ablation catheter. Entrance and exit blocks were validated in the SP group by a conventional CMC-based analysis in the first 11 patients (19%, phase I) and in the last 16 patients (28%, phase III). Antral entrance and exit blocks identified by a CF-guided ablation catheter and PVI validated by a CMC catheter (checking for entrance and exit block) were concordant in 91 of 92 assessed PVs. Hence, the positive predictive value of the presence of antral entrance and exit blocks using solely the ablation catheter reached 99% for PVI (Table 3).

**Table 3:** Operator learning curve (only control ablation protocol group, n = 57)

	**Phase I (*n* = 11)**	**Phase II (*n* = 30)**	**Phase III (*n* = 16)**	***p* value**
RF time PVI (sec, median [IQR])	2373 [2178, 2932]	1892 [1665, 2105]	1920 [1620, 2043]	**0.002**
Fluoro time (min, median [IQR])	2 [1, 3]	2 [1, 3]	2 [1, 3]	0.887
Fluoro dose (Gycm^2^, median [IQR])	237 [57, 528]	240 [110, 438]	170 [70, 269]	0.621
Procedure duration (min, median [IQR])	125 [116, 176]	122 [105, 140]	110 [102, 120]	0.058
Stick to map time (min, median [IQR])	24 [16, 29]	21 [14, 30]	16 [13, 24]	0.401
Map duration (min, median [IQR])	16 [14, 18]	19 [17, 23]	16 [14, 8]	**0.029**
RF duration (min, median [IQR])	97 [76, 106]	74 [64, 83]	72 [70, 81]	0.104
Number of lesions	102 [79, 120]	85 [77, 109]	79 [71, 89]	0.151
First pass Isolation (%)	6 (60)	26 (90)	16 (100)	0.01
CTI (%)	3 (27)	9 (30)	2 (12)	0.411
RF time for CTI (sec, median [IQR])	294 [287, 442]	303 [254, 368]	419 [318, 520]	0.972
Overall RF time (sec, median [IQR])	2620 [2358, 3016]	1976 [1891, 2206]	1962 [1620, 2135]	**0.003**
Energy dose (Joules, median [IQR])	68,091 [56070, 75356]	53,724 [47292, 57406]	53,360 [43757, 57658]	0.069
***Outcome data***				0.964
Recurrence during FU (%)	2 (18)	5 (17)	2 (14)	0.264
Redo procedures (%)	0 (0)	4 (67)	1 (50)	0.13

Data are presented as *n* (%) or median (IQR). Abbreviations: *IQR* interquartile range; *RF* radiofrequency energy; *Fluoro* fluoroscopy; *CTI* cavotricuspid isthmus ablation; *FU* follow-up

### First-pass isolation

**Fig. 3:**
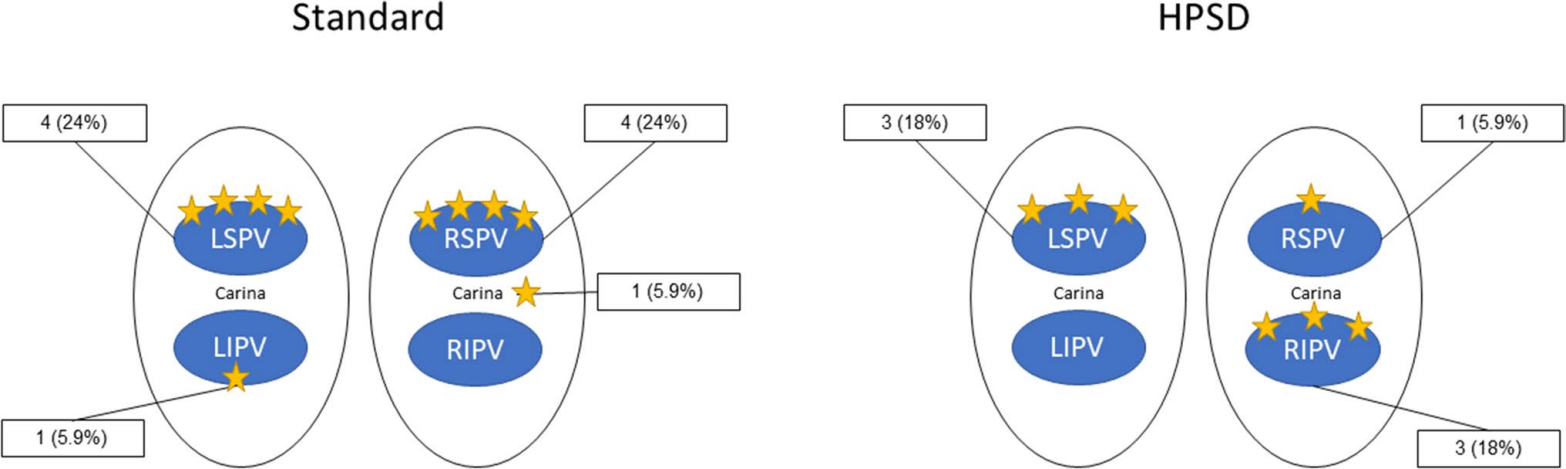
Location of conduction gaps for control ablation protocol vs. HPSD ablation-index guided protocol. Conduction gaps are illustrated for the control single-catheter RF ablation approach (left) and the high-power short-duration (HPSD) ablation index–guided protocol (right) in patients in whom first-pass isolation was not achieved. There were a total of 17 conduction gaps. Each conduction gap is represented by a yellow star

In 78 (88%) cases, first-pass isolation (FPI) of the PVs was achieved after initial encirclement of PV antra. FPI PVI was achieved using the HPSD ablation index–guided protocol in 33 patients (88%) and using the SP in 48 patients (87%), *p* = 1.0. Of the 13 patients without FPI, 7 (54%) had only one gap, while 6 patients (46%) had a maximum of two gaps. A total of 17 residual gaps were detected. The most frequent location requiring touch-up ablations was the LSPV in 41% of cases and the RSPV Carina in 30% of cases. Distribution of conduction gaps stratified for the SP group versus the HPSD group is illustrated in Fig. 3. Mapping with the ablation catheter alone was successful in identifying these gaps. Antral exit block was achieved with a median additional RF time of 215 s (IQR 111–313) in all cases.

### Follow-up

In this study, there were no major complications related to the procedures. The median follow-up of the study was 336 days (IQR 174–386). Arrhythmia recurrence following a 3-month blanking period after a single procedure was seen in 12 (18%) patients. At 12 months, Kaplan–Meier survival analysis showed 87% of patients free from atrial arrhythmias in the study. The rates of freedom from AT/AF at 1 year were similar between the HPSD ablation-index guided protocol group and the SP group (91% vs. 84%, *p* = 0.67). There were no serious complications during follow-up.

## Discussion

The purpose of this study was to compare the efficacy, efficiency, and safety of a minimalistic, streamlined single-catheter RF ablation approach using a HPSD ablation index–guided protocol with a control single-catheter RF ablation protocol for index PVI in patients with AF.

Our main findings are as follows: First, using a single-catheter RF ablation approach is an effective, efficient, and safe approach for first-time ablation in patients with paroxysmal or persistent AF. When using a HPSD protocol, there is a significant reduction in procedural duration and RF time compared to the SP with the same excellent rate of freedom of RF at 1 year. Second, using an ablation-index guided single-catheter protocol, first-pass isolation is achieved in the majority of patients, both with HPSD and in the SP group. The residual gap is identifiable with the ablation catheter in all cases. Third, median fluoroscopy time and median radiation exposure were minimal in both groups thereby minimizing radiation exposure to the patient, physician, and staff. Thus, a low-radiation workflow not using fluoroscopy after transseptal puncture [23] is feasible using a single-catheter protocol in the vast majority of patients. Fourth, the implementation of a single-catheter technique was associated with a learning curve. The improved operator skills resulted in a significant reduction in the procedure and RF duration over time.

To the best of our knowledge, the present study compares for the first time the single-catheter approach using a HPSD ablation index–guided protocol vs. a SP. The success rate, which was close to 90%, was similar to that reported by several studies in patients with paroxysmal undergoing ablation with contact force sensing technology or virtual efficacy parameters [8, 10, 12, 13]. Single-catheter RF ablation protocols have been evaluated for the remote magnetic navigation system [24]. Vollmann reported a higher PV isolation rate using the CMC compared to the group without CMC. In a Belgian single-center retrospective analysis, material costs, procedure time, and radiation exposure were reduced in the single-catheter protocol compared with the CMC group. Freedom of recurrence was similar between groups [19]. Pambrun et al. [17] assessed the feasibility of single-catheter PVI with CF-sensing catheters using a standard ablation protocol (30 W, 25 W for the posterior wall) and compared it to the use with a CMC. They found that CF-guided single-catheter ablation achieved successful acute PVI in 98% of the study group and a 31% reduction in estimated cost. This study did not focus on cost-effectiveness, but the reported approach is effective, associated with short procedure times and omitting the CMC is cost-saving.

Gupta et al. [18] extended the single-catheter protocol using CF-sensing catheters to an AI-guided CLOSE protocol approach. Adherence to the CLOSE protocol with a single-catheter setup yielded a near 90% freedom from atrial arrhythmias at 1-year follow-up similar to the clinical outcome in our series. In our study, the median procedure time in the HPSD ablation-index guided protocol group was 84 min (IQR 76–100). This is comparable to single-shot balloon techniques for AF ablation, ranging from 89 to 139 min in two recent studies [4, 5]. This reduction in procedure duration in our study compared to a CMC-guided PVI is most likely explained by the absence of the second transseptal puncture, the abandonment of the CMC catheter and using a HPSD ablation-index guided protocol.

The proposed workflow may translate into increased clinical safety: limiting the number of transseptal punctures may reduce the risk of tamponade. Since only one femoral access is required, the risk of groin complications such as AV fistula or groin hematoma is reduced. The risk of stroke or bleeding might be expected to be lower by reducing catheter time in the LA and the potential of cerebral embolism should be lower when avoiding exchanging CMC with ablation catheter through a single transseptal sheath. Another potential safety benefit of a HPSD approach might be the lower risk of deep penetration of conductive heating to adjacent structures such as the esophagus or the phrenic nerve [25], which needs to be clinically validated in larger studies. Finally, omitting the CMC eliminates the risk of entanglement in the mitral valve apparatus.

Cardiac biomarkers have been used to estimate myocardial injury after catheter ablation of AF using different energy sources and catheters [26, 27]. hs-cTn has been proposed to provide comparative information regarding magnitude of myocardial injury and endocardial lesion size; however, the clinical significance is unknown. Whether more myocardial injury detected by hs-cTn might worsen the heading damage of adjacent structures remains unknown. In this study, no significant difference in the clinical safety profile has been observed between both groups. However, given the infrequency of such complications, these results must be interpreted with caution until larger, multicenter trials can corroborate these findings. Depending of the financial compensation model of the healthcare system, the cost of the procedure can be reduced to different degrees. All these aforementioned measures (no CMC catheter, single transseptal puncture, reduced procedure time) are likely also cost saving. In addition, this workflow does not routinely entail an intracardiac echocardiography catheter, no general anesthesia and no esophageal temperature probe, which will likely yield additional reduction of costs. To improve healthcare utilization, increased efficiency is an accepted goal as long as procedural safety and effectiveness are not diminished. Therefore, we believe that this very standardized and simplified approach to PVI is very reasonable for first time AF ablation procedures. Novel technology allowing even shorter RF applications with very high power such as the QDOT MICRO (Biosense Webster) catheter will further help accelerate this transformation [28]. This catheter contains micro-electrodes highlighting only local potentials and not far-field potentials, thereby potentially improving detection of near-field PV potentials post ablation compared to normal bipolar EGMs.

Procedural characteristics improved over time in our study. Several techniques allowed to optimize the workflow. For precise delimitation of the veno-atrial junction, we used the “swing-fall” technique as previously described [19]. Regarding testing for PVI isolation, the operator has to be familiar with several pitfalls: First, to avoid poor catheter-tissue contact when testing for exit block and to carefully assess for local capture, the force-vector orientation displayed on the Carto3 system is helpful (see Fig. 3, central illustration). Second, far-field potentials from the LA appendage or superior vena cava can be differentiated by the morphology of the local EGM, by reducing pacing output and by assessing timing measurements from the local EGM to the beginning of the surface P-wave. Importantly, residual PV gaps were present in a minority of patients after initial delivery of PV and the number of residual gaps after first circumferential PVI significantly decreased over time. Regarding quick and precise localization of residual gaps, we recommend assessing for residual conduction along the intervenous carinal tissue and anterior ridge of the LSPV, which accounted for 48% of all gaps in this cohort. In the remaining cases, assessing earliest timing of the PVP in sinus rhythm is usually sufficient to identify residual conduction gaps. The ablation catheter is usually easily maneuverable around the PV antra and thus provides the optimal catheter set-up to check for isolation.

## Limitations

This study should be interpreted in light of certain methodologic limitations. First, this is a retrospective analysis of a prospective database with all ensuing limitations. While the segmentation of the standard group in different phases might help to estimate the impact of the learning curve on procedural efficacy, it does not completely overcome this methodological issue. Second, patients were not randomized to either ablation protocol. However, baseline characteristics were matched well between groups. Third, this is a single-center study, and reproducibility should be analyzed in a multicentric fashion. Fourth, for the HPSD protocol power was set to 35 W at the posterior wall and 50 W at the anterior wall. We did not assess higher power settings such as 60–90 W or higher power settings for the posterior wall. Further research is required to ascertain the optimal power and duration that conveys the maximum potential clinical benefit with the least possible risk. Fifth, several technical novelties could have increased the safety and efficacy of a HPSD approach such as use of esophageal monitoring [29] or protection devices [30], use of a steerable, visualized sheath [31] or use of a contact force-sensing catheter optimized for temperature-controlled RF ablation [28]. Sixth, this study only used a point-by-point technique. A comparison between single-shot devices and current state-of-the-art point-by-point technique using HPSD should be evaluated in a randomized setting.

## Conclusion

In conclusion, a minimalistic, CMC-free HPSD-guided PVI approach is very efficient, safe, likely cost-saving, and associated with excellent clinical outcomes at 1 year. Prospective studies are required to confirm the reproducibility of the outcomes across a wider range of centers.

## Supplementary Information

Below is the link to the electronic supplementary material.

Supplementary file1 (DOCX 15 KB)
