# Supplementary material for: High-power short-duration ablation index–guided pulmonary vein isolation protocol using a single catheter
Source: J Interv Card Electrophysiol. 2022 May 20;65(3):633–42. doi: 10.1007/s10840-022-01226-9 (PMC9726791; doi:10.1007/s10840-022-01226-9)
Supplement: Supplementary file 1 — Supplementary file1 (DOCX 15 KB) [file 10840_2022_1226_MOESM1_ESM.docx]

**Online Supplemental Appendix**

- **Table S1:** Procedural characteristics with excluded patients from the training phase (n=11)

| **Table S1** | **Procedural characteristics with excluded patients from the training phase (n=11)** | | | | |
| --- | --- | --- | --- | --- | --- |
|  | | **All patients**  **(n=80)** | **Control protocol**  **(n=46)** | **HPSD protocol**  **(n=34)** | **p-Value** |
| RF time PVI (sec, median [IQR]) | | 1598 [1094, 1945] | 1896 [1656, 2100] | 1036 [898, 1184] | **<0.001** |
| Fluoro time (min, median [IQR]) | | 2 [1, 3] | 2 [1, 3] | 2 [1, 3] | 0.637 |
| Fluoro dose (Gycm^2^, median [IQR]) | | 163 [76, 307] | 210 [89, 355] | 149 [69, 205] | 0.201 |
| Procedure duration (min, median [IQR]) | | 104 [83, 119] | 116 [103, 137] | 84 [76, 100] | **<0.001** |
| Stick to map time (min, median [IQR]) | | 18 [13, 26] | 19 [13, 28] | 18 [14, 22] | 0.323 |
| Map duration (min, median [IQR]) | | 16 [12, 21] | 18 [15, 22] | 12 [10, 16] | **<0.001** |
| RF duration (min, median [IQR]) | | 66 [46, 77] | 74 [65, 83] | 44 [35, 57] | **<0.001** |
| Number of lesions | | 78 [67, 88] | 84 [75, 95] | 67 [60, 79] | **<0.001** |
| First pass Isolation (%) | | 51 (66) | 28 (61) | 23 (74) | 0.334 |
| CTI ablation (%) | | 19 (24) | 11 (24) | 8 (24) | 1 |
| RF time for CTI (sec, median [IQR]) | | 322 [228, 514] | 303 [247, 492] | 335 [209, 501] | 0.741 |
| Overall RF time (sec, median [IQR]) | | 1620 [1161, 1996] | 1976 [1690, 2164] | 1107 [934, 1329] | **<0.001** |
| Energy dose (Joules, median [IQR]) | | 51170 [41795, 57029] | 53724 [45009, 57406] | 43312 [37484, 53156] | **0.002** |
| hs-cTn before RF (ng/L, median [IQR]) | | 7 [5, 11] | 7 [5, 11] | 7 [6, 11] | 0.476 |
| hs-cTn after RF (ng/L, median [IQR]) | | 868 [699, 1159] | 828 [636, 1023] | 996 [724, 1264] | **0.037** |
| ***Outcome data*** | |  |  |  |  |
| Recurrence during FU (%) | | 12 (13) | 9 (16) | 3 (8.8) | 0.669 |
| Redo procedures (%) | | 5 (5.5) | 5 (8.8) | 0 (0) | 1 |

Data are presented as n (%) or median (IQR). Abbreviations: IQR, interquartile range; RF, radiofrequency enegery; Fluoro, fluoroscopy; CTI, cavotricuspid isthmus ablation; FU, follow-up.
